# The association of rs1051730 genotype on adherence to and consumption of prescribed nicotine replacement therapy dose during a smoking cessation attempt

**DOI:** 10.1016/j.drugalcdep.2015.03.035

**Published:** 2015-06-01

**Authors:** Jennifer J. Ware, Paul Aveyard, Peter Broderick, Richard S. Houlston, Timothy Eisen, Marcus R. Munafò

**Affiliations:** a MRC Integrative Epidemiology Unit (IEU) at the University of Bristol, BS8 2BN Bristol, United Kingdom; b School of Social and Community Medicine, Oakfield House, Oakfield Grove, Bristol BS8 2BN, United Kingdom; c Nuffield Department of Primary Care Health Sciences, University of Oxford, Oxford OX2 6GG, United Kingdom; d Division of Genetics and Epidemiology, Institute of Cancer Research, Sutton, Surrey SM2 5NG, United Kingdom; e Cambridge University Health Partners, Addenbrookes Hospital, Cambridge CB2 0QQ, United Kingdom; f UK Centre for Tobacco and Alcohol Studies and School of Experimental Psychology, University of Bristol, 12a Priory Road, Bristol BS8 1TU, United Kingdom

**Keywords:** Smoking, Nicotine replacement therapy, Cessation, Genetics

## Abstract

**Introduction:**

While nicotine replacement therapy (NRT) is an effective pharmacological smoking cessation treatment, its efficacy is influenced by adherence to and consumption of the prescribed dose. The genetic variant rs1051730 in the nicotinic receptor gene cluster *CHRNA5-A3-B4* influences smoking quantity. The aim of this study was to explore the impact of rs1051730 genotype on adherence to and consumption of NRT prescription following a smoking cessation attempt.

**Methods:**

Secondary analysis of data from a pharmacogenetic smoking cessation trial. Participants (*n* = 448) were prescribed a daily dose of NRT for four weeks post quit attempt, and monitored during weekly clinic visits. Outcome measures were NRT prescription adherence rate (%) and average daily NRT consumption (mg) at 7- and 28-days after the quit attempt.

**Results:**

An association between rs1051730 genotype and both outcome measures was observed at 7-days after the quit date. Each copy of the minor allele corresponded to a 2.9% decrease in adherence to prescribed NRT dose (*P* = 0.044), and a 1.0 mg decrease in daily NRT consumption (*P* = 0.026). Adjusting for number of cigarettes smoked during this period only slightly attenuated these associations. There was no clear statistical evidence of an association between genotype and adherence or consumption at 28-days.

**Conclusions:**

This is the first study to evaluate the impact of rs1051730 genotype on consumption of and adherence to NRT prescription during a smoking cessation attempt. We observed an association between this variant and both outcome measures at 7-days; however, this was only moderate. These findings require replication in an independent sample.

## Highlights

• We explore the impact of rs1051730 on nicotine replacement therapy (NRT) use in a smoking cessation trial.
• Outcomes were NRT prescription adherence rate (%) and daily consumption (mg).
• rs1051730 was associated with both outcomes at 7 days post quit attempt.
• Each copy of the minor allele corresponded to a 1 mg decrease in daily NRT consumption.
• No evidence of effect of genotype on either outcome observed at 28 days.

## 1 Introduction

Nicotine replacement therapy (NRT) is an effective pharmacological smoking cessation treatment relative to placebo (Cahill et al., 2013). The efficacy of NRT is influenced by a number of factors, including type of NRT used, and combination versus single type use (Cahill et al., 2013). Adherence to and consumption of NRT prescription also influences efficacy; Shiffman (2007) showed that the use of more NRT lozenges was associated with better smoking cessation outcomes—each additional lozenge consumed was associated with increased odds of quitting success by 10%. Maximising treatment adherence and consumption is likely to maximise treatment efficacy, and identifying factors that influence this may therefore be valuable.

Several factors underlie differences in adherence to and consumption of NRT, such as the form in which it is prescribed (Hajek et al., 1999; Hollands et al., 2013). Genetic factors may also influence adherence to and consumption of NRT. One genetic locus relevant to smoking behaviours lies within the nicotinic acetylcholine receptor subunit gene cluster *CHRNA5-A3-B4* on chromosome 15. Variants rs1051730 in *CHRNA3* and rs16969968 in *CHRNA5* (which are almost perfectly correlated in European populations and therefore essentially interchangeable) robustly associate with nicotine dependence, self-reported and objective assessments of smoking quantity (e.g., cotinine levels), and several smoking-related diseases, the minor allele conferring increased risk (Munafo et al., 2012; Thorgeirsson et al., 2008; Ware et al., 2012). Notably, rs16969968 is a missense mutation, resulting in an amino acid change (aspartate to asparagine) in the resultant alpha-5 nicotinic receptor subunit protein. This change is associated with reduced receptor function in vitro (Bierut et al., 2008). Preclinical evidence suggests that that the alpha-5 nicotinic receptor subunit, encoded by *CHRNA5*, influences self-titrated nicotine exposure via tolerance to the toxic effects of high doses of nicotine (Fowler et al., 2011). There is also evidence that this variant is associated with a reduced likelihood of smoking cessation (Munafo et al., 2011; Taylor et al., 2014), and reduced body mass index (BMI) in individuals who smoke (Freathy et al., 2011).

Identifying genetic variants associated with adherence to and consumption of NRT would be valuable for a number of reasons. First, it may provide insight into individual differences in the use of NRT, and inform treatment strategies. Second, it might afford genetic variants suitable for use as instrumental variables in Mendelian randomisation analyses of large, existing datasets (Ware and Munafo, 2014). This latter approach, in particular, would enable the efficient exploration of the consequences of adherence and consumption on a range of outcomes. Therefore, using data from a smoking cessation trial, we explored the impact of rs1051730 genotype on adherence to and consumption of an NRT prescription following a smoking cessation attempt.

## 2 Materials and methods

### 2.1 Design and overview

We conducted a secondary analysis of data from a randomised controlled trial investigating the impact of informing smokers that their prescribed NRT dose had been tailored according to genotype (*OPRM1*) or phenotype (nicotine dependence). Full details of this trial have been reported previously (Marteau et al., 2012, 2010). Ethics approval for this trial was granted by the Hertfordshire Research Ethics Committee (06/Q0201/21). Informed written consent was obtained from all study participants.

### 2.2 Participants

Participants were adult smokers who had consumed at least 10 cigarettes per day over the preceding 12 months, and were motivated to quit. Cigar, pipe and oral tobacco users who did not also smoke 10 or more cigarettes per day were excluded. Additional exclusion criteria included contraindication for NRT use, previous severe adverse reactions to NRT patch or oral NRT, current use of smoking cessation medication, and current use of medication for another indication with a known influence on smoking cessation (e.g., nortriptyline). Non-English speakers and those deemed unsuitable for the study by their primary care physicians were also excluded. The trial took place in smoking cessation clinics in primary care.

### 2.3 Procedure

All participants were offered behavioural support and an NRT prescription. Behavioural support was offered twice prior to quit day and weekly thereafter for four weeks. An additional session was offered eight weeks after the quit date. NRT consisted of a nicotine patch, the strength of which was based on the participant's daily cigarette consumption (10–14 cigarettes per day = 14 mg; ≥15 cigarettes per day = 21 mg). In addition, participants were also prescribed a second, oral ‘top-up’ form of NRT (gum, lozenge, sublingual tablet or inhalator), which delivered 6 or 12 mg of *absorbed* nicotine. This ‘top-up’ dosage was based on *OPRM1* genotype or level of nicotine dependence (determined by the Fagerström Test for Nicotine Dependence; Heatherton et al., 1991) depending on which treatment arm of the trial the participant had been randomised to. NRT was prescribed for four weeks from the quit date.

Participants attended seven weekly clinic sessions. Baseline measures were assessed during the first clinic session. NRT was prescribed and a quit date agreed during the second session. The quit attempt commenced after the third session. Participants were requested to take their NRT as prescribed for four weeks post quit attempt and to attend weekly clinic sessions, where NRT adherence checks were performed and repeat prescriptions administered. Information regarding use of cigarettes during the preceding week was also collected during clinic sessions.

### 2.4 Genotyping

DNA was extracted from EDTA-venous blood samples and Picogreen quantified (Invitrogen). Genotyping of rs1051730 was conducted by competitive allele-specific PCR KASPar chemistry (LGC, Hertfordshire, UK) implemented on an ABI7900HT platform (Applied Biosystems, Foster City, USA) (details available on request).

### 2.5 Outcome measures

*Adherence to prescription of NRT*: Adherence was defined as the proportion (%) of all NRT prescribed that was consumed on each day, averaged over 7 and 28 days after the quit date, and was assessed by the clinic nurse during weekly clinic visits. Assessments were based on participant diaries and a count of remaining NRT.

*Daily NRT consumption*: Consumption was defined as the dose of nicotine consumed on each day (mg), averaged over 7 and 28 days after the quit date, calculated using total prescribed daily dose (i.e., 20, 26, 27 or 33 mg) and relevant adherence rate.

### 2.6 Statistical plan

Linear regression was used to assess the relationship between rs1051730 genotype and NRT adherence and consumption outcomes. Genotype was coded additively according to the number of minor alleles (0, 1, 2) and treated as a continuous variable. Unadjusted, partially adjusted, and fully adjusted models were tested for each outcome at 7- and 28-day time points. The partially adjusted model adjusted for age, sex, socioeconomic position, trial condition (i.e., genotype or phenotype arm), baseline BMI and baseline daily cigarette consumption. The fully adjusted model additionally adjusted for number of cigarettes smoked on top of NRT prescription during the specified period.

Given the previously observed association between rs1051730 and short-term smoking cessation success (Munafo et al., 2011), where we observed an association between genotype and NRT adherence and consumption outcomes, we re-ran these analyses limiting our sample to those individuals who were abstinent. This was done so as to rule out smoking relapse as a potential explanation for any association observed between genotype and outcome measures. At the 7-day time point, abstainers were defined as those reporting smoking fewer than 5 cigarettes in the first week after the quit date (*n* = 315), while at the 28-day time point abstainers were defined using Russell standard procedures (West et al., 2005) as those reporting smoking fewer than 5 cigarettes in the past two weeks, verified by an exhaled carbon monoxide reading of less than 10 ppm, and counting participants lost to follow up as being smokers (*n* = 245).

With the sample available for analysis (*n* = 448), assuming a minor allele frequency of 0.35 and an additive model of inheritance, we calculated that we had 80% power to detect a 4.1% (7 days) and 5.8% (28 days) reduction in NRT adherence per minor allele, and a 1.3 mg (7 days) and 1.7 mg (28 days) reduction in NRT consumption per minor allele at an alpha level of 5%. We also examined the relationship between rs1051730 genotype and smoking abstinence at 7 and 28 days (as defined above) using logistic regression. A *χ*^2^ test was used to assess whether genotype frequencies deviated from Hardy-Weinberg equilibrium. All analyses were conducted in SPSS version 21 (IBM Corp., Armonk, NY).

## 3 Results

### 3.1 Participant selection

Of the 633 individuals who were randomised in the full trial, we limited our analyses to those of European ancestry with rs1051730 data and NRT prescription adherence rates >0% at 7 days. This resulted in a final sample size of 448.

### 3.2 Participant demographics

Participant demographic information and baseline measures are presented for the full sample and by rs1051730 genotype in Table 1. Genotype frequencies for rs1051730 did not deviate substantially from Hardy-Weinberg equilibrium (*P* = 0.06).

### 3.3 Impact of rs1051730 on adherence to NRT prescription

An association between rs1051730 genotype and adherence to NRT prescription was observed at 7 days after the quit attempt (see Table 2). Each copy of the minor allele corresponded to a 2.9% decrease in adherence to prescribed NRT dose over this time period (95% CI −5.6% to −0.1%, *P* = 0.044). This association was robust to adjustments made for age, sex, socioeconomic status, trial condition, baseline BMI and baseline daily cigarette consumption (−3.0%, 95% CI −5.8% to −0.1%, *P* = 0.043). Additionally adjusting for number of cigarettes smoked in addition to NRT during this period only slightly attenuated this association (−2.4%, 95% CI −4.9% to 0.1%, *P* = 0.061). Limiting the sample to those individuals who reported smoking fewer than 5 cigarettes in the first week had little impact on this association (unadjusted [*n* = 315]: −3.0%, 95% CI −5.5% to −0.5%, *P* = 0.021; partially adjusted [*n* = 306]: −3.0%, 95% CI −5.6% to −0.3%, *P* = 0.027; fully adjusted [*n* = 306]: −3.0%, 95% CI −5.6% to −0.4%, *P* = 0.026). No association between genotype and adherence was observed at 28-days after the quit attempt (*P*s > 0.61 for unadjusted, partially adjusted and fully adjusted analyses).

### 3.4 Impact of rs1051730 on NRT consumption

An association between rs1051730 genotype and average daily NRT consumption was observed at 7 days after the quit attempt (see Table 2). Each copy of the minor allele corresponded to a 1.0 mg decrease in mean daily NRT consumption over this time period (95% CIs −1.8 mg to −0.1 mg, *P* = 0.026) (for reference, mean daily NRT consumption for the full sample within this time period was 22.9 mg). This association was robust to adjustments made for age, sex, socioeconomic status, trial condition, baseline BMI and baseline daily cigarette consumption (−1.0 mg, 95% CIs −1.9 mg to −0.2 mg, *P* = 0.021). Additionally adjusting for number of cigarettes smoked on top of NRT during this period had little impact this effect (−0.9 mg, 95% CIs −1.6 mg to −0.1 mg, *P* = 0.022). A similar association was observed when the sample was limited to those individuals who reported smoking fewer than 5 cigarettes in the first week (unadjusted [*n* = 315]: −1.0 mg, 95% CIs −1.9 mg to −0.2 mg, *P* = 0.020; partially adjusted [*n* = 306]: −0.9 mg, 95% CIs −1.7 mg to 0.0 mg, *P* = 0.039; fully adjusted [*n* = 306]: −0.9 mg, 95% CIs −1.7 mg to 0.0 mg, *P* = 0.039). No association between genotype and NRT consumption was observed at 28 days (*P*s > 0.36 for unadjusted, partially adjusted and fully adjusted analyses).

### 3.5 Impact of rs1051730 on abstinence

There was no evidence of association between rs1051730 and likelihood of abstinence at 7 days (OR = 1.10, 95% CIs 0.81 to 1.51, *P* = 0.55) or 28 days (OR = 0.89, 95% CIs 0.68 to 1.16, *P* = 0.39). These results were not altered substantially when adjusted for age, sex, socioeconomic position, trial condition, baseline BMI and baseline daily cigarette consumption.

## 4 Discussion

To our knowledge this is the first study to explore the association of rs1051730 genotype on consumption of and adherence to NRT prescription during a smoking cessation attempt. We observed an association with both outcome measures at 7 days after the quit attempt, each copy of the minor allele conferring a 2.9% decrease in adherence to prescribed NRT dose, and a 1.0 mg decrease in average daily NRT consumption. However, no clear evidence of association of rs1051730 with either outcome was observed at 28 days. Understanding the factors that underlie adherence to and consumption of NRT is of clinical importance given the likely impact of actual NRT consumption on smoking cessation outcomes. Using data from the same trial, Hollands et al. (2013) found that the odds of 28 day abstinence increased by 5% for each additional 1 mg of NRT consumed per day.

Previous research has demonstrated a robust association between rs1051730 and measures of smoking quantity, both self-reported and objective; the minor allele is associated with higher indices of smoking quantity. Given this, one might hypothesise that the minor allele would be associated with increased adherence to and consumption of prescribed NRT, in an effort to achieve an adequate nicotine dose to alleviate withdrawal. However, the association observed between genotype and adherence/consumption outcomes at 7 days was in the opposite direction. Moreover, the association observed was only mildly attenuated by adjustment for baseline daily cigarette consumption and cigarettes smoked on top of NRT prescription during the specified period. It is worth noting that any association of genotype with adherence/consumption may be mediated via tolerance to nicotine. If so, adjustment for cigarette consumption may in fact lead to adjustment for this mediating mechanism. The fact that this adjustment did not alter our results substantially indicates that nicotine tolerance may not in fact be the relevant mediating mechanism. However, given the known plasticity of smoking behaviour this adjustment is likely to be imperfect (McNeill and Munafo, 2013; Munafo et al., 2012), which precludes drawing strong conclusions from the relatively little change that this adjustment made to our results.

One potential explanation for the effect we observed is that rs1051730 minor allele carriers were more likely to abandon their quit attempt and treatment prior to the end of the trial. This is certainly plausible; the rs1051730 minor allele was associated with reduced likelihood of cessation success at 4 weeks in open-label NRT study (Munafo et al., 2011). If minor allele carriers were abandoning their quit attempt and hence treatment during the 7-day period, this would result in an association between rs1051730 genotype and NRT adherence/consumption; that is, the minor allele would be associated with reduced adherence and consumption, as we observed. To explore this issue, we re-ran the 7-day analyses limiting the sample to those individuals who reported smoking fewer than 5 cigarettes in the first week post quit. However, we observed the same association in this sub-sample, which is inconsistent with this explanation. Further, no association was observed between rs1051730 and smoking abstinence at either time-point, but this may have been due to lack of statistical power, and the odds ratio at 28 days was comparable to previously observations (Munafo et al., 2011).

Two study limitations are worthy of note. First, consumption of and adherence to NRT prescription were determined from participant diaries and counts of remaining medication during clinic sessions. Cotinine levels would have provided a more precise, objective assessment of these outcomes. Second, our sample was small for a genetic association study, and a replication sample was not available. These findings should therefore be considered preliminary and hypothesis-generating until replicated independently.

Using data from a smoking cessation trial, we observed an association between rs1051730 genotype and adherence to and consumption of NRT prescription. Each copy of the minor allele was associated with decreased consumption and adherence at 7 days after the quit attempt, although no association of genotype and consumption or adherence was observed at 28 days. However, given the weak statistical evidence for association, our findings should be interpreted with caution, and replication in an independent sample sought.

## Author disclosures

### Role of funding source

This study was funded as part of a grant from the Medical Research Council (Risk communication in preventive medicine: Optimising the impact of DNA risk information; G0500274 PI: Theresa Marteau). Support from the Medical Research Council (MC_UU_12013/6) is gratefully acknowledged. The work of PB and RSH is supported by a grant from Cancer Research UK (C1298/A8362). JJW is supported by a Post-Doctoral Research Fellowship from the Oak Foundation. JJW, PA and MRM are members of the UK Centre for Tobacco and Alcohol Studies, a UK Clinical Research Council Public Health Research: Centre of Excellence. Funding from British Heart Foundation, Cancer Research UK, Economic and Social Research Council, Medical Research Council, and the National Institute for Health Research, under the auspices of the UK Clinical Research Collaboration, is gratefully acknowledged. The funding sources had no involvement in study design, in the collection, analysis and interpretation of data, in the writing of the report, or in the decision to submit the article for publication.

## Contributors

MRM conceived the study design. JJW and MRM analysed the data. All authors contributed to the interpretation of the results. All authors contributing to drafting the paper or critically revising it, and all read and approved the final submission.

## Conflict of interest

MRM and TE have received grant support from Pfizer, who manufacture smoking cessation products. PA has done consultancy and research for manufacturers of smoking cessation medication. TE has taken part in advisory boards for and received honoraria from GSK, which manufactures smoking cessation products. TE is on part-time leave of absence from the University of Cambridge to work with AstraZeneca.

## Figures and Tables

**Table 1:** Demographic characteristics and baseline measures.

	Total sample	rs1051730 genotype			
		GG	GA	AA	*P*-value
Total sample (*N*, %)	448 (100%)	201 (43.0%)	184 (39.4%)	63 (13.5%)	N/A

Age (M, SD)	48.2 (12.9)	49.8 (12.6)	47.5 (13.1)	45.3 (12.7)	0.031

FTND (M, SD)	5.6 (2.2)	5.7 (2.1)	5.5 (2.3)	5.6 (2.1)	0.497

Cigarettes/Day (M, SD)	21.1 (8.5)	21.2 (8.1)	20.9 (8.7)	21.5 (9.5)	0.873

Sex (*N*, %)					
Male	205 (45.8%)	84 (41.8%)	90 (48.9%)	31 (49.2%)	0.314
Female	243 (54.2%)	117 (58.2%)	94 (51.1%)	32 (50.8%)	

BMI (M, SD)	27.2 (5.6)	27.1 (5.4)	27.5 (6.1)	26.3 (4.8)	0.336

SES (*N*, %)					
Low	130 (29.0%)	65 (32.3%)	49 (26.6%)	16 (25.4%)	0.035
Medium	156 (34.8%)	57 (28.4%)	68 (37.0%)	31 (49.2%)	
High	162 (36.2%)	79 (39.3%)	67 (36.4%)	16 (25.4%)	

Trial condition (*N*, %)					
Genotype	226 (50.4%)	102 (50.7%)	90 (48.9%)	34 (54.0%)	0.782
Phenotype	222 (49.6%)	99 (49.3%)	94 (51.1%)	29 (46.0%)	

NRT Prescription (*N*, %)					
20 mg	76 (17.0%)	29 (14.4%)	37 (20.1%)	10 (15.9%)	0.518
26 mg	7 (1.6%)	4 (2.0%)	3 (1.6%)	0 (0.0%)	
27 mg	281 (62.7%)	130 (64.7%)	107 (58.2%)	44 (69.8%)	
33 mg	84 (18.8%)	38 (18.9%)	37 (20.1%)	9 (14.3%)	

*P*-values generated using chi-square test for categorical variables and one-way ANOVAs for continuous variables. M = mean; SD = standard deviation; BMI = body mass index; SES = socioeconomic status; FTND = Fagerström test for nicotine dependence; NRT = nicotine replacement therapy. All analyses based on sample of 448 individuals with the exception of BMI which was based on total sample of 435 (11 individuals with missing data, 2 individuals with severely outlying BMI values [>100]).

**Table 2:** The impact of rs1051730 genotype on adherence to prescribed NRT dose and actual NRT consumption following a quit attempt.

Outcome	Unadjusted			Partially adjusted			Fully adjusted		
	*N*	B (95% CI)	*P*-value	*N*	B (95% CI)	*P*-value	*N*	B (95% CI)	*P*-value
7 days									
Adherence	448	−2.856 (−5.631 to −0.082)	0.044	435	−2.967 (−5.844 to −0.090)	0.043	413	−2.372 (−4.853 to 0.108)	0.061
Consumption	448	−0.970 (−1.823 to −0.116)	0.026	435	−1.003 (−1.851 to −0.155)	0.021	413	−0.865 (−1.606 to −0.124)	0.022

28 days									
Adherence	448	−0.990 (−4.882 to 2.902)	0.617	435	−0.680 (−4.669 to 3.309)	0.738	294	0.150 (−2.045 to 2.344)	0.893
Consumption	448	−0.522 (−1.648 to 0.603)	0.362	435	−0.427 (−1.574 to 0.720)	0.465	294	−0.151 (−0.895 to 0.593)	0.690

Beta estimates reflect change in NRT adherence (%) or daily NRT consumption (mg) per copy of the rs1051730 A allele. Partially adjusted model includes age, sex, socioeconomic position, trial condition (i.e., top-up prescription based on genotype or phenotype), baseline daily cigarette consumption, and baseline BMI. Fully adjusted model further adjusts for number of cigarettes smoked on top of NRT prescription during the specified period.
